# Effectiveness of the Power Dry-Land Training Programmes in Youth Swimmers

**DOI:** 10.2478/v10078-012-0025-5

**Published:** 2012-05-30

**Authors:** Jerzy Sadowski, Andrzej Mastalerz, Wilhelm Gromisz, Tomasz NiŸnikowski

**Affiliations:** 1 Jozef Pilsudski University of Physical Education, Warsaw, Department of Physical Education and Sport, Biała Podlaska, Poland.

**Keywords:** ergometer, strength, tethered force, youth swimmers

## Abstract

The aim of the study was to evaluate the effects of the dry-land power training on swimming force, swimming performance and strength in youth swimmers. Twenty six male swimmers, free from injuries and training regularly at least 6 times a week, were enrolled in the study and randomly assigned to one of two groups: experimental (n=14, mean age 14.0 ± 0.5 yrs, mean height 1.67±0.08 m and mean body mass 55.71 ±9.55 kg) and control (n=12, mean age 14.1 ± 0.5 yrs, mean height 1.61±0.11 m and mean body mass 49.07 ±8.25 kg). The experimental group took part in a combined swimming and dry-land power training. The control group took part in swimming training only. The training programmes in water included a dominant aerobic work in front crawl. In this research the experimental group tended to present slightly greater improvements in sprint performance. However, the stroke frequency insignificantly decreased (−4.30%, p>0.05) in the experimental group and increased (6.28%, p>0.05) in the control group. The distance per stroke insignificantly increased in the experimental group (5.98%, p>0.05) and insignificantly decreased in the control group (−5.36%, p>0.05). A significant improvement of tethered swimming force for the experimental group (9.64%, p<0.02) was found, whereas the increase was not statistically significant in the control group (2.86%, p>0.05). The main data cannot clearly state that power training allowed an enhancement in swimming performance, although a tendency to improve swimming performance in tethered swimming was noticed.

## Introduction

Power training has the potential to develop muscle strength under dynamic conditions. Power represents the ability to perform movements at high speed or the possibility of exerting high strength in a short period of time. The relationship between the power, strength and speed of muscle contraction was first described by Hill more than sixty years ago (1938). Swimming performance is a multifactorial phenomenon involving energetics, biomechanics, hydrodynamics, anthropometrics and strength parameters (Barbosa et al., 2008; 2010).

Strength and speed are major factors determining performance of swimmers (Trappe and Pearson, 1994). Scientists and coaches agree that training should include both land and water training sessions. Strength and endurance training in swimming takes place both on land and in the water. Several studies showed that a combination of strength and endurance training inhibit strength and power development (Dudley and Djamil, 1985; Abernethy and Quigley, 1993; Hennessey and Watson, 1994). However, inconclusive results are presented in scientific literature. Several studies showed that concurrent strength and endurance training increases the development of strength and power (Dudley and Djamil, 1985; Hennessey and Watson, 1994), and other studies reported that concurrent training has an inhibitory effect on the development of strength and endurance (Sale et al., 1990; Abernethy and Quigley, 1993).

Studies including dry-land training reported positive effects on sprint performances in swimming (Costill, 1999; Pichon et al., 1995; Strass, 1988). Nevertheless, Tanaka et al. (1993) did not find performance enhancement after a dry-land strength training period. These authors stated that combined swimming and traditional dry-land strength training did not improve swimming performance, whereas combined swimming strength and swimming-specific in the water strength training increased swimming velocity. Tanaka et al. (1993) claim that strength exercises executed in the water are more efficient than dry-land training. Several studies support the view that strength dry-land training in swimming improves swimming performance (Girold et al., 2006; 2007; Toussaint and Vervoorn, 1990; Trappe and Pearson, 1994) and performance-related parameters such as increased stroke length (Toussaint and Vervoorn, 1990), reduced stroke rate (Girold et al., 2006; 2007) and increased tethered swimming force (Girold et al., 2006; 2007; Toussaint and Vervoorn, 1990; Trappe and Pearson, 1994). Dry-land training includes specially structured strength training program performed usually with the resistance of the swimmer body. More often training sessions are assisted by specific apparatus including ergometers for swimming paddling as well as cycle ergometers. These devices allow to improve the swimming performance in spite of the swimming drag. They offer an alternative for overloading an athlete especially under maximal velocity conditions. The ability to perform movements with high speed is represented by muscle power. Power is also characterized by potential opportunities to develop muscle force in dynamic conditions. The differences between training based on power and force were described by Häkkinen et al. (1985). Primarily power training should improve the regulatory mechanisms between neural and muscle systems. Muscle power is the product of muscle force and action velocity, each of which is influenced by intrinsic muscle properties (Moritani, 2003). Bigland-Ritchie (1981) suggests that short-term power training might have been brought about by the neural adaptations in terms of greater muscle activation levels and more synchronous activation patterns. Neural adaptations may cause beneficial effects on work economy (Hoff et al., 2002). Rouard et al. (1992) showed that muscular recruitment was higher for maximal swimming speed than slow speed. In order to achieve such effects, it is necessary to use an ergometer which provides an imitation of the underwater phase of the upper or lower limb.

Hence, while studies including strength training protocols under dry-land condition reported positive effects on sprint performances in swimming (Costill, 1999; Pichon et al., 1995; Strass, 1988), it seems important to analyze the effects of power training on swimming performance in youth swimmers. Therefore, the aim of the study was to estimate the effect of dry-land power training on swimming force, swimming performance and strength in youth swimmers. In this study, the power training consisted of specially designed resistance force training performed under dynamic conditions. The maximum velocity of motion during power training was controlled by varying external resistance to obtain maximum power.

## Material and Methods

### Subjects

Twenty six male swimmers, free from injuries and training regularly at least 6 times a week, took part in the research. They were randomly assigned to one of the two groups: experimental (n=14, mean age 14.0±0.5 yrs, mean height 1.67±0.08 m and mean body mass 55.71±9.55 kg) and control (n=12, mean age 14.1 ± 0.5 yrs, mean height 1.61±0.11 m and mean body mass 49.07±8.25 kg). The experimental group was engaged in a combined programme of swimming and dry-land power training. The control group engaged in swimming training only. The subjects provided written informed consent to participate in this research, and the procedures were approved by the institutional review board and University Ethics Committee. The swimmers in the experimental group consisted of stroke specialists in freestyle (seven persons), butterfly (three), breaststroke (two) and individual medley (two). Two of them were sprinters, the rest were middle or long distance swimmers. The control group consisted of 6 freestyle, two breaststroke, one backstroke and three butterfly swimmers. One of them was a sprinter.

### Training device

**Figure 1:**
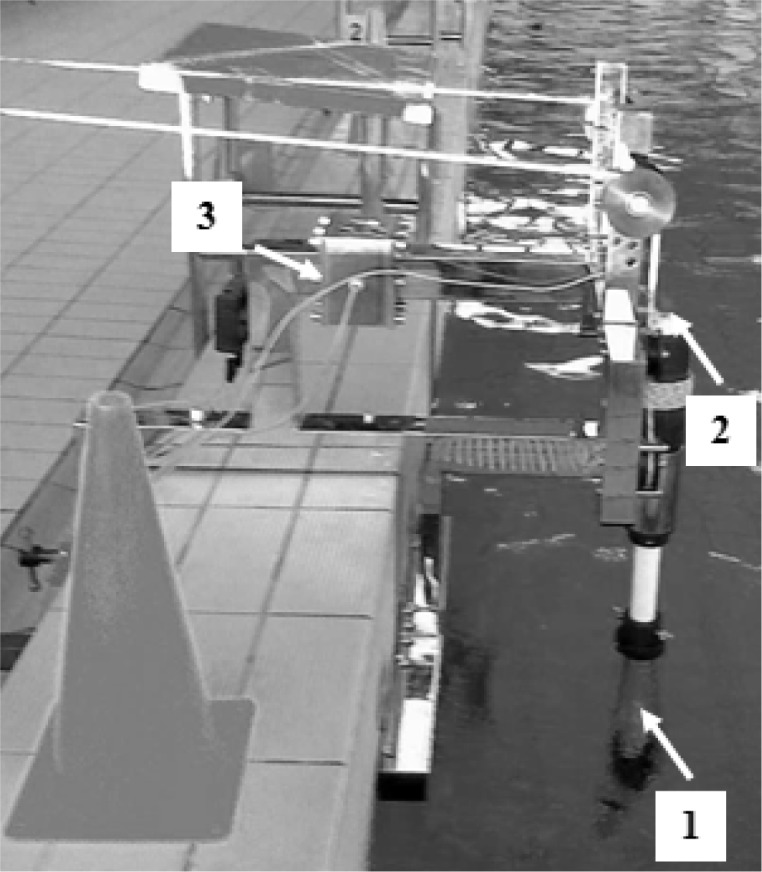
*The ergometer applied during the experiment*.

A “hydroisokinetic” ergometer (Sadowski et al., 2005) was applied during the power training sessions (Figure 1). This ergometer simulates the underwater phase of shoulder work during the front crawl. The ergometer has a base frame made of stainless steel with a screw mechanism for mounting on the edge of a swimming pool. During training, each subject lay prone on a bench, assuming the same body position held during swimming..

The tested subject drove the ergometer by holding handles connected to a rotary head equipped with blades with a freely adjustable geometry (1). During the exercise session, the forces and stroke lengths of the motions of the right and left arm were measured. A two-component force transducer (3) was used to estimate the force during control measurements and the training sessions

The length of each stroke was measured using a potentiometer (2) located along the axis of the rotating head.

### Training procedure

#### Swimming training

Swimming training sessions were held six times a week (from Monday to Saturday). The training was performed in a 25 m pool according to the methodology described by Brems (1984). Before each training session, the swimmers performed a warm-up for 10–20 minutes. The training program lasted from February to March, and represented the second macrocycle of the season. Swimmers were preparing for their regional championships that were to take place in April. The swimming training program lasted for six weeks (59 training sessions). Swimmers practiced twice a day (6^45^ – 8^15^ and 14^00^ – 15^30^) every day of the week excluding Sundays. One morning training was performed on Saturday (7^30^ – 9^00^). Average training volume and intensity were the same for all swimmers throughout the study protocol. The training program included a dominant aerobic work in the front crawl. Detailed analysis of swimming training is presented in Table 1 and 2.

All subjects swam over in total the distance of 273.50 km during the whole experiment. To determine the training intensity the heart rate was controlled for every one swimming distance presented in Table 1.

#### Design of power training

In the experimental group, the power-training programme on land using the ergometer lasted six weeks (18 sessions). Training sessions were held three times a week (Monday, Wednesday and Friday) before training in the water, preceded by a 10-minute warm-up. Training sessions using the ergometer consisted of 6 sets of 50 seconds of work with 10 seconds rest intervals.

Previous results for swimmers with different levels of performance indicate that the optimal frequency of swimming cycles during competition ranges from 51 to 60 strokes/minute, and the main influence on performance is the length of each cycle (Maglischo, 2003). Therefore, during each training session directed at power development, we observed the frequency of the swim cycles and determined the resistance accordingly. We assumed that the applied resistance was ineffective when the movements using the ergometer exceeded 60 strokes/minute. To increase exercise intensity, the geometry of the blades in the water was adjusted as needed to obtain an effective level of resistance.

### Test procedures

Control measurements were conducted before the start (pretest) of the experiment and after six weeks of combined swimming and dry-land power training (post-test). These measurements consisted of the following assessments:

- the assessment of isometric strength (IS),
- the assessment of swimming performance during the front crawl driven by the upper extremities over 25 m (V25),
- the assessment of strength during tethered swimming (TS).

Both the experimental and control groups were evaluated at the same time. The evaluations were conducted over one week in each evaluation period.

The measurement of isometric strength (IS) was conducted on the ergometer (Figure 1). Two 5-second measurements of shoulder flexion with arms adjusted at the angles of 45° between arms and trunk were performed. The greatest value of IS was chosen for analysis. The signals were captured at 400 Hz by a computer interface and stored in a data acquisition program for later analysis.

One day after IS tests, the swimmers performed a 25 m free style time trial. Stroke length and stroke frequency were determined with a camera. Speed of locomotion was defined as a product of the stroke rate SR and distance per stroke SD and can be defined through the time of stroke cycle T:

(1) $$V=\frac{\mathrm{SD}}{T}=\frac{SD\cdot \mathrm{SR}}{60}$$

The equation (1) for the distance per stroke (SD) is presented below:

(2) $$\mathrm{SD}=V\cdot T=\frac{60\cdot V}{\mathrm{SR}}$$

The tethered swimming force was determined in two incremental tests with the swimmers connected to a 1,000 N load cell with 4 strain gauges attached with commercial elastic cord (StrechCordz Long Belt Slider (12–31 lb), NZ Manufacturing, Inc., USA). Strength was evaluated in tests over 10 seconds (TS). The signals were captured at 400 Hz by a computer interface and stored in the data acquisition program.

### Statistical analysis

The normality of the distributions was checked with Shapiro-Wilk tests (StatSoft, Inc., 2007) using the STATISTICA data analysis software system (version 8.0., www.statsoft.com), and non-normal distributions were found. All differences between groups were evaluated by Mann-Whitney tests, and within-group differences between pre and post-training were assessed by Wilcoxon tests.

## Results

### Strength performance

**Figure 2:**
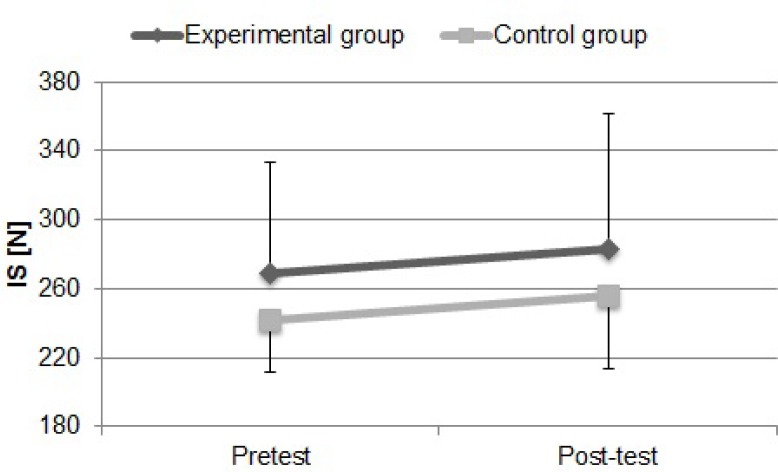
Shoulder-strength values during isometric test (IS) determined for the experimental and control group during initial and final evaluations

Figure 2 presents the values of isometric force obtained during the shoulder flexion on the ergometer, for the experimental and the control group during the initial and final evaluations.

Force during shoulder flexion determined during the IS test insignificantly increased by 5.34% (p>0.05) between pretest and post-test in the experimental group as well as in the control group (5.69%, p>0.05). After six weeks of power training the control group still presented higher values than the experimental group in the IS test (10.83%), but the difference between both group decreased insignificantly by 0.38%.

### Swimming performance

**Figure 3:**
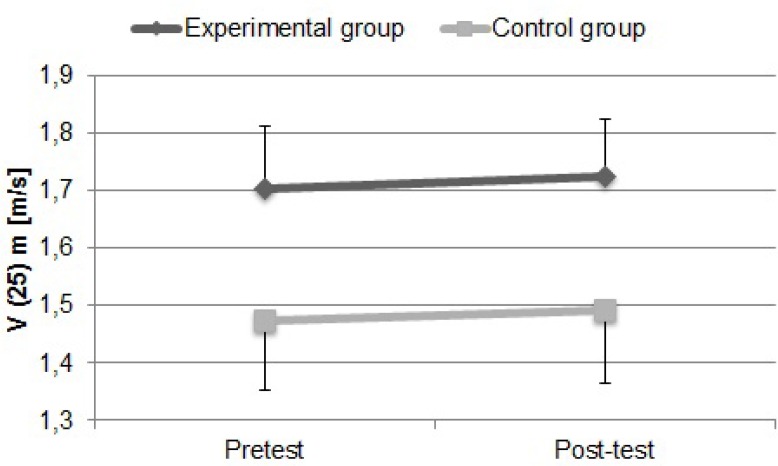
Swimming performance in 25 m front crawl at the beginning of the protocol (Pretest) and after six weeks of training (Post-test) for the experimental and control group

Figure 3 presents the swimming performance in the 25 m front crawl, at the beginning of the protocol (pretest) and after six weeks of training (post-test).

Significant differences were registered between the experimental and the control group during the initial and final evaluations (15.58%, p<0.001 and 15.70%, p<0.001 - respectively for pretest and post-test). The experimental group showed slightly greater improvements in sprint performance. The experimental group insignificantly increased the performance from pretest to post-test by 1.30% (p>0.05), as well as an insignificant increase in performance was also observed from pretest to post-test conditions (1.16%, p>0.05) in the control group.

**Figure 4:**
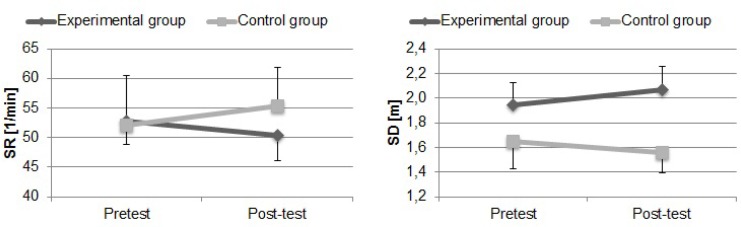
Mean and standard deviation values for stroke frequency (SR) and distance per stroke (SD) in the 25 m front crawl at the beginning of the protocol (Pretest) and after six weeks of training (Post-test) for the experimental and control groups

The data for stroke frequency and stroke distance during swimming performance in the 25 m front crawl for two groups are presented in Figure 4. Stroke frequency insignificantly decreased (−4.30%, p>0.05) in the experimental group and increased (6.28%, p>0.05) in the control group.

Additionally, significant differences were registered between the experimental and control group at the post-test (8.92%, p<0.03). The distance per stroke insignificantly increased in the experimental group (5.98%, p>0.05) and insignificantly decreased in the control group (−5.36%, p>0.05). Moreover, significant differences were observed between the experimental and control group during the two measurements (18.20%, p<0.001 and 32.34%, p<0.001 -respectively for pretest and post-test).

### Strength in tethered swimming

A significant improvement of tethered swimming force for the experimental group (9.64%, p<0.02) was found, whereas the increase was not statistically significant from pretest to post-test conditions (2.86%, p>0.05) in the control group. In addition, tethered swimming force was similar in the control and experimental groups at pretest and post-test evaluations (p>0.05). Moreover, the experimental group presented higher values of TS compared with the control group (35.50%, p<0.001) after six weeks of power training. However, the experimental group presented higher values of TS compared with the control group (27.10%, p<0.002) from the start of the research.

## Discussion

This study aimed to investigate the effects of a six week combined dry-land strength and aerobic swimming training on upper body strength, tethered swimming force and swimming performance in youth competitive swimmers. It is considered that traditional dry-land strength training or combined swimming and strength training do not appear to enhance swimming performance in untrained individuals or competitive swimmers (Tanaka and Swensen, 1998). It was previously observed that although combined training increases upper body strength, it does not result in faster sprint times compared with swimming-only training (Tanaka et al., 1993). Additionally, Bulgakova et al. (1990) reported that water strength training was more effective than dry-land strength exercises. Additionally, Bulgakova et al. (1990) reported that strength training in water is more effective than dry-land strength training. Many authors indicate that in youth swimmers technical training is more important than strength training (Kjendlie et al., 2004, Aspenes et al. 2009; Barbosa et al., 2010; Garrido et al., 2010). According to our data, it is not clear that strength training allowed the improvement in swimming performance in youth swimmers although a tendency to improve performance due to both types of training was noticed. It is uncertain whether dry-land strength training leads to improved sprint performance while swimming. Tanaka et al. (1993) explained lack of significant transfer between dry-land strength training and swim performance by the ambiguity of swimming and dry-land training condition. Different factors determine to what extent training progress in one situation leads to improved performance in the other. Previous studies showed that training effects are angle and velocity-specific. This suggests that effects of dry-land strength training are transferred to the swimming if during strength training people move just as fast or even faster than during swimming in water (Toussaint et al., 1988). Moreover, not only the movement velocity during strength training should correspond to that during swimming, but also the relationship between joint angle and strength must be similar to that in water condition.

In our research the experimental group showed a slightly greater improvement in sprint performance (Figure 3). However, the stroke frequency insignificantly decreased (−4.30%, p>0.05) in the experimental group and increased (6.68%, p>0.05) in the control group. The explanation for this could be the reason that swim specific frequencies during dry-land and muscle load are different from the movement pattern in the water (Bollens et al., 1988; Toussaint et al., 1988). Several studies on dry-land trainings reported positive effects on sprint performances in swimming and generally they observed improvements between 1.3 and 4.4% (Costill, 1999; Pichon et al., 1995; Strass, 1988).The specific dry-land power training applied in that research did not influence on the distance per stroke. The distance per stroke insignificantly increased in the experimental group (5.98%, p<0.04) as well as in the control group (3.33%, p>0.05). The improvement of tethered swimming force was as expected, and this training effect is in line with the study by Girold et al. (2007). Our results are in line with the study by Aspenes et al. (2009). In this study, the experimental group improved dry-land strength, tethered swimming force and 400 m freestyle performance more than the control group. No changes were observed in stroke length, stroke rate, and performance in 50 and 100 m freestyle.

Several prior studies demonstrated strong relationships between upper body strength and 25 m and 50 m sprint swimming performance (Sharp et al., 1982; Hawley et al., 1992; Smith et al., 2000). Unfortunately, power training applied in our research in the experimental group improved the upper body strength and swimming performance insignificantly. In other studies investigating the effects of dry-land strength training on swimming (Tanaka et al., 1993; Trappe and Pearson, 1994; Girold et al., 2007) only Girold et al. (2007) found benefits of combined strength and swimming training. The improvement of swimming performance depends on the specificity of the training methods (Tanaka and Swensen, 1998; Costill, 1999; Stewart and Hopkins, 2000) and training intensity (Mujika et al., 1995; Chatard and Mujika, 1999). In our study, the dry-land power training program was adopted as specifically as possible to the water conditions. Unfortunately, the imitation of the underwater phase of shoulder work in the front crawl provided by the ergometer in a combined program of swimming and dry-land power training cannot be used as the useful training method applied in youth swimmers. For practitioners, the investigation may be useful in determining ways to optimize training especially in context of power training. The main data cannot clearly state that power training allowed an enhancement in swimming performance, although a tendency to improve swimming performance in tethered swimming, due to the power training was noticed.

## Figures and Tables

**Figure 5:**
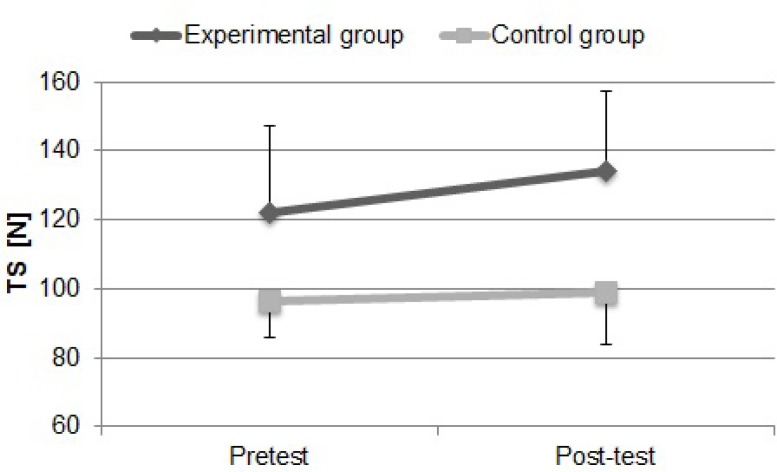
Tethered swimming force over periods of 10 seconds (TS) during the swimming test at the beginning of the protocol (Pretest) and after six weeks of training (Post-test) for the experimental and control groups

**Table. 1:** Characteristics of the energy zones and their application to the training (percentage rates shown in parentheses)

	**Aerobic 2, EN1**	**Mix 3, EN2–3**	**Anaerobic 4, SP1–2**	**Sprint 5, SP3**
**Distance [km]**	226.21 (82.71)	35.50 (12.98)	7.44 (2.72)	4.35 (1.59)
**Heart rate [bpm]**	120 – 145	145 – 175	175 and more	-
**Duration of exercise**	12 min and more	3 – 12 min	10 sec – 3 min	0 – 15 sec
**Swimming distance [m]**	1500 – 3000	400 – 1200	100 – 200	15 – 50

**Table. 2:** Training volume in relation to the swimming styles (percentage rates shown in parentheses)

	**Front crawl**	**Freestyle**	**Backstroke**	**Butterfly**	**Breaststroke**	**Arms**	**Legs**	**Technique**
**Distance [km]**	24.92 (68.16)	19.46 (53.22)	2.75 (7.52)	10.45 (28.58)	2.78 (7.60)	14.35 (39.25)	9.99 (27.32)	15.30 (41.85)
